# Senescent cardiomyocytes contribute to cardiac dysfunction following myocardial infarction

**DOI:** 10.21203/rs.3.rs-2776501/v1

**Published:** 2023-04-10

**Authors:** Rachael Redgrave, Emily Dookun, Laura Booth, Omowumi Folaranm, Simon Tual-Chalot, Jason Gill, Andrew Owens, Ioakim Spyridopoulos, Joao Passos, Gavin Richardson

**Affiliations:** Newcastle University; Newcastle University; Newcastle University; Newcastle University; Newcastle University; Newcastle University; Newcastle University; Newcastle University; Mayo Clinic; Newcastle University

## Abstract

Myocardial infarction is a leading cause of morbidity and mortality. While reperfusion is now standard therapy, pathological remodeling leading to heart failure remains a clinical problem. Cellular senescence has been shown to contribute to disease pathophysiology and treatment with the senolytic navitoclax attenuates inflammation, reduces adverse myocardial remodeling and results in improved functional recovery. However, it remains unclear which senescent cell populations contribute to these processes. To identify whether senescent cardiomyocytes contribute to disease pathophysiology post-myocardial infarction, we established a transgenic model in which p16 (CDKN2A) expression was specifically knocked-out in the cardiomyocyte population. Following myocardial infarction, mice lacking cardiomyocyte p16 expression demonstrated no difference in cardiomyocyte hypertrophy but exhibited improved cardiac function and significantly reduced scar size in comparison to control animals. This data demonstrates that senescent cardiomyocytes participate in pathological myocardial remodeling. Importantly, inhibition of cardiomyocyte senescence led to reduced senescence-associated inflammation and decreased senescence-associated markers within other myocardial lineages, consistent with the hypothesis that cardiomyocytes promote pathological remodeling by spreading senescence to other cell-types. Collectively this study presents a novel demonstration that senescent cardiomyocytes are major contributors to myocardial remodeling and dysfunction following a myocardial infarction. Therefore, to maximize the potential for clinical translation, it is important to further understand the mechanisms underlying cardiomyocyte senescence and how to optimize senolytic strategies to target this cell lineage.

## Introduction, Results, Discussion

Myocardial infarction (MI), is the leading cause of death and disability in developed countries.^1^ Even, with reperfusion therapy, pathological myocardial remodelling can impact patient health by progressively impairing cardiac function, leading to heart failure.^2^ Several independent studies have shown that MI causes senescence in numerous myocardial cell types, including cardiomyocytes (CMs), fibroblasts and endothelial cells^3,4^ (reviewed;^5,6^). Post-MI treatment with mechanistically diverse senolytic compounds, reduces senescent cell number, decreases inflammation, and improves heart function, suggesting that senescence contributes to disease pathophysiology.^4,7^ Although the role of fibroblast senescence has been studied in this disease setting^8^ it remains unclear if senescent CMs actively participate in disease pathophysiology. Indeed, it has been proposed that as CMs lack a meaningful regenerative capacity,^9,10^ their elimination could in fact be detrimental and senotherapies improve outcomes despite, rather than as a result of CMs apoptosis.^6,7^ Moreover, while senolytic treatment improves outcome following MI, studies have failed to address the possibility that the observed benefits are due to non-myocardial senescent cell elimination or peripheral off-target effects.^6,7^ For example, the molecular pathways influenced by senolytics are not uniquely expressed by senescent cells^11^ and non-resident myocardial cell populations which contribute to remodelling, including T-lymphocytes^12,13^ and platelets,^14^ are influenced by senolytic treatment.^15,16^ To continue the development of effective senotherapies, it’s imperative that the contribution of individual senescent cell populations to disease progression is understood.

**Figure 1:**
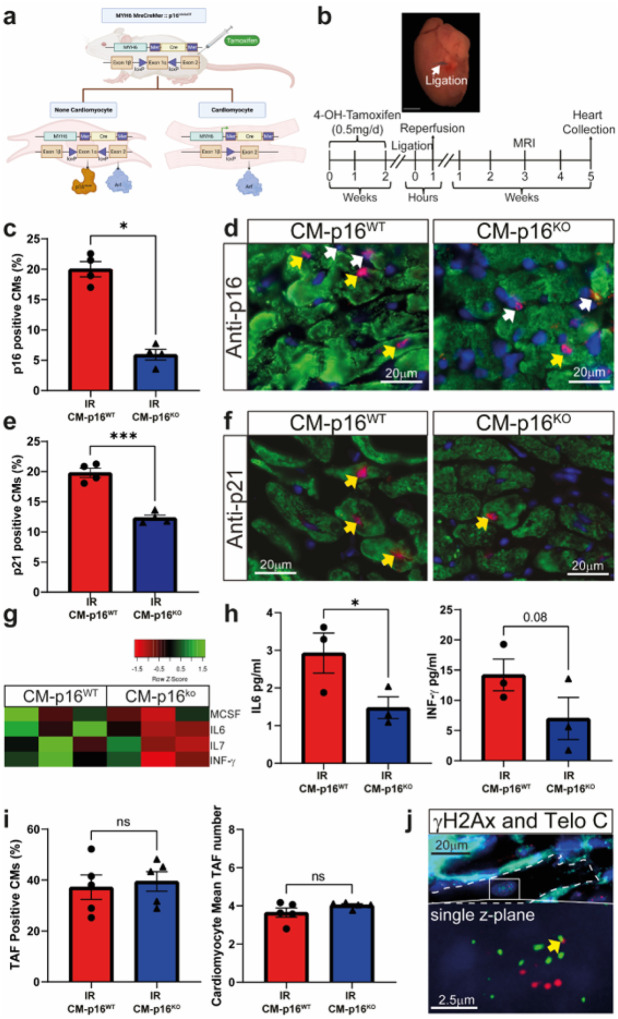
Cardiomyocyte specific knock out of p16 reduces cardiomyocyte senescence and senescence associated secretory phenotype. **(a)** schematic of transgenic cardiomyocyte p16 knock-out mouse **(b)** Experimental design. **(c)** Percentage p16^+^ CMs in the peri-infarct region at 5 weeks post IR. **(d)** Representative images of p16 staining. Yellow arrow, p16^+^ CM and white arrow p16^+^ interstitial cell (p16 red, Troponin-C green, DAPI blue), n=4/group. **(e)** Percentage p21^+^ CMs in the peri-infarct region at 5 weeks post IR. **(f)** Representative images of p21 staining. Yellow arrows, p21^+^ CM (p21 red, Troponin-C green, DAPI blue), n=4 per group. **(g)** Clustered heatmap of cytokine protein levels in LV myocardium. **(h)** Expression of individual protein levels in the LV myocardium of heart in the indicated experimental groups. **(i)** Total number of cardiomyocytes with TAF and mean of TAF per cardiomyocyte at 5 weeks post IR. n= 3/group. **(j)** Representative images of TAF, γH2AX co-localized with Telomere immuno-FISH (telo-FISH red, γH2AX green, WGA white). Images are obtained from the z-stacks of 10μm sections. Yellow arrow indicates a TAF. Scale bars as indicated. Data are mean±SEM, *P<0.05 , ***P<0.05 using Student T-test or Mann Whitney test.

The cyclin dependant kinase inhibitor p16 plays a key role in regulating CM senescence. p16 is increased in CMs with age and in response to myocardial infarction.^4,17^ Furthermore, in aged *INK-ATTAC* mice, pharmacogenetic clearance of p16, reduces the percentage of CMs expressing alternative markers of senescence, reducing CM hypertrophy, and improving heart function.^17^ Therefore, to investigate the specific role of CM senescence in disease pathophysiology following MI with reperfusion we employed a transgenic model that allows the CM specific inactivation of *CDKN2A* (which encodes p16).^4^ CM-p16^**KO**^ and CM-p16^**WT**^ mice were subjected to cardiac ischaemia reperfusion (IR) and assessed 5 weeks post-surgery (Fig. 1a and b; supplementary experimental procedures). No animals died post-surgically in either group, and no differences in weight were observed between groups both pre- and post-IR (**Supplementary Fig. 1**). At 5 weeks the peri-infarct region of CM-p16^**KO**^ mice contained significantly fewer p16-expressing CMs compared to CM-p16^**WT**^ controls, indicating that p16 was effectively knocked out in CMs (Fig. 1c and d). CMs expressing p21 (a senescence marker) were also reduced in the peri-infarct region of CM-p16^**KO**^ mice compared to CM-p16^**WT**^, but to a lesser extent than p16 (Fig. 1e and f). Additionally, a decrease in senescence associated secretory phenotype (SASP) proteins with described roles in myocardial remodelling^18^ was also observed in the myocardium of p16-CM^**KO**^ mice (Fig. 1g and h). IR induces telomere foci of DNA damage (TAF) within CMs and TAF can trigger activation of p16 and p21 associated senescence pathways.^17^ As would be expected given that TAF induction is upstream of p16 activation there was no difference in mean TAF number or the percentage of CMs with TAF between the two groups. This is also suggestive that the degree of insult and stress as a result of IR was similar between the two groups (Fig. 1i and j). These data indicate that, while p16 independent senescence may still occur in the CMs following inactivation of p16^**INK4**^, CM senescence was dampened in the CM-p16^**KO**^ model.

**Figure 2:**
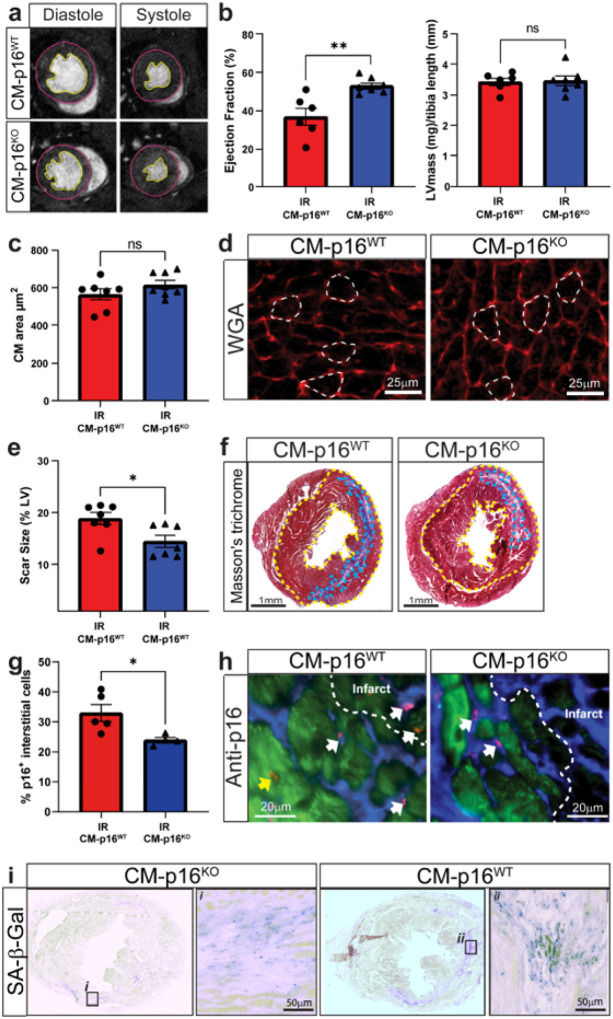
Cardiomyocyte specific inhibition of senescence improves functional outcome, reduces scar size and attenuates senescence in interstitial cells following IR. **(a)** Examples of individual short axis cine-MR images. **(b)** Ejection fraction and LV mass at 5 weeks post-IR, n>6/group. **(c)** Mean CM cross-sectional area μm^2^. N=7/group. **(d)** Representative images of WGA staining for quantification of CM area. **(e)** Quantification of infarct size relative to total LV area. N=7/group. **(f)** Representative images of Masson’s trichrome staining. **(g)** Percentage p16^+^ interstitial cells in the peri-infarct region at 5 weeks post IR. **(h)** Representative images of p16 staining. Yellow arrows, p16^+^ CMs and white arrows, p16^+^ interstitial cells (p16 red, Troponin-C green, DAPI blue), n=4/group. **(i)** Representative image of SA-β-gal staining at 5 weeks post IR. Scale bars as indicated. Data are mean±SEM,;; * P<0.05, **P<0.01using students T-test or Mann Whitney Test.

To determine if senescent CMs contribute to maladaptive myocardial remodelling, and therefore impaired cardiac function after IR, the mice were analysed using cardiac magnetic resonance imaging (MRI) (Fig. 2a and b). At 5 weeks post-IR CM-p16^**KO**^ mice had a significantly higher ejection fraction compared to CM-16^**WT**^ littermates (Fig. 2b). This was attributed to improved preservation of left ventricular (LV) systolic volume, as no significant difference in LV end diastolic volume was observed between the groups. A trend in improved stroke volume was also evident in CM-p16^**KO**^ mice (**Supplementary Fig. 2**). Senescence has been previously linked to pathological CM hypertrophy.^17,19^ We therefore aimed to identify if improved maintenance of cardiac function was a result of the attenuation of pathological hypertrophy in the CM-16^**KO**^ hearts. No differences were observed in hypertrophy measured at an organ or cellular level as LV mass indexed to tibia length and mean CM area were consistent between experimental groups (Fig. 2b, c and d). This data suggests that, in the acute setting, senescence is not a leading driver of hypertrophy and alternative pathways, for example the angiotensin II and the renin-angiotensin-aldosterone system pathways may be more important.^20^ Interestingly, scar size was significantly reduced in CM-p16^**KO**^ mice, suggesting that CM senescence contributes to this aspect of pathological remodelling after IR (**Figure** e **and f**). This data together with the observed reduction in SASP expression in the myocardium of p16-CM^**KO**^ mice led us to hypothesise that CM senescence and SASP has a paracrine influence on surrounding non-CM cell populations. Supporting this hypothesis, CM-p16^**KO**^ mice demonstrated a significant reduction in p16 expressing interstitial cells and reduced senescence associated-β-galactosidase staining in the myofibroblast rich area of infarct at 5 weeks post IR (Fig. 2g, h and i). Furthermore, studies have demonstrated that senescence induction promotes myofibroblast differentiation and enhances collagen deposition.^8,21^ This, together with our current study provides a mechanism by which senescent CMs promote scar formation and cardiac dysfunction following IR.

Collectively, this study demonstrates for the first time, that following IR, CM senescence is detrimental to cardiac function and promotes myocardial remodelling, adding to the growing body of evidence which suggests that post-mitotic cell senescence plays a crucial role in the pathophysiology of various diseases. The data also indicates that this is a result of SASP expression, which induces paracrine senescence and promotes fibrosis and scar production. This suggests a more in-depth study of CM SASP could uncover unique biomarkers that are more accurate in predicting myocardial dysfunction compared to SASP from other cell types found across multiple organ systems. In conclusion, it is crucial that future therapeutic approaches consider the significant contribution of senescent CMs to myocardial remodelling and dysfunction.

## Methods

All methods are included in supplementary experimental procedures.

## Data Availability

All data generated or analysed during this study are included in this published article and its supplementary information files.
